# Dynamic Bayesian Model for Detecting Obstructive Respiratory Events by Using an Experimental Model †

**DOI:** 10.3390/s23073371

**Published:** 2023-03-23

**Authors:** Daniel Romero, Raimon Jané

**Affiliations:** 1 ESAII Department, Universitat Politècnica de Catalunya—BarcelonaTech (UPC), 08019 Barcelona, Spain; 2 Institute for Bioengineering of Catalonia (IBEC-BIST), 08028 Barcelona, Spain; 3 CIBER of Bioengineering, Biomaterials and Nanomedicine (CIBER-BBN), 28029 Madrid, Spain

**Keywords:** obstructive sleep apnea, probabilistic models, respiratory events, chronic respiratory diseases

## Abstract

In this study, we propose a model-based tool for the detection of obstructive apnea episodes by using ECG features from a single lead channel. Several sequences of recurrent apnea were provoked in separate 15-min periods in anesthetized rats during an experimental model of obstructive sleep apnea (OSA). Morphology-based ECG markers and the beat-to-beat interval (RR) were assessed in each sequence. These markers were used to train dynamic Bayesian networks (DBN) with different orders and feature combinations to find a good tradeoff between network complexity and apnea-detection performance. By using a filtering approach, the resulting DBNs were used to infer the apnea probability signal for subsequent episodes in the same rat. These signals were then processed using by 15-s epochs to determine whether epochs were classified as apneic or nonapneic. Our results showed that fifth-order models provided suitable RMSE values, since higher order models become significantly more complex and present worse generalization. A global threshold of 0.2 gave the best overall performance for all combinations tested, with Acc = 81.3%, Se = 69.8% and Sp = 81.5%, using only two parameters including the *RR* and Ds (R-wave downslope) markers. We concluded that multivariate models using DBNs represent a powerful tool for detecting obstructive apnea episodes in short segments, which may also serve to estimate the number of total events in a given time period.

## 1. Introduction

Recurrent apnea during a patient’s sleep is caused by repeated episodes of obstructive sleep apnea (OSA), resulting in prolonged exposure to intermittent hypoxia (IH). This chronic condition has been associated with some cardiovascular consequences, including among others, systemic hypertension, heart failure, coronary artery disease, and stroke [1,2,3]. At the same time, OSA patients usually present excessive daytime sleepiness and nonrestorative sleep, fatigue, impaired learning, and significant social problems due to poor mental performance.

Nowadays, overnight polysomnography (PSG) is considered the gold standard for OSA diagnosis and detection, which includes multiple physiological signals such as electroencephalogram (EEG), electrooculogram (EOG), electrocardiogram (ECG), electromyogram (EMG), and blood oxygen level (SpO${}_{2}$), among others [1]. However, analysis of PSG recordings is very expensive, time consuming, and uncomfortable for the patient, limiting its accessibility and suitability for long-term home monitoring or ambulatory systems [2,3,4]. Recent efforts have been made to propose lighter systems with few sensors or a single channel, but they focus mainly on signal acquisition [5,6,7]. Likewise, other systems that analyze signals in real time often have insufficient accuracy for medical diagnosis [8]. However, in recent years many promising algorithms for offline OSA detection and diagnosis have been implemented with proven performance, showing great potential for their implementation on lighter hardware and wearable devices [9].

A variety of algorithms typically use a set of features extracted from PSG recordings and apply machine learning, neural network, or deep learning techniques for OSA detection or classification as reported in [9]. Among them, some use a single-source sensor for SpO${}_{2}$ [10,11,12], respiration [13,14,15], or sound [16,17,18] for this purpose. Others, however, use ECG channels and have been demostrated to achieve the highest classification accuracy [9], followed by oxygen saturation, respiration, and sound-based methods. The ECG-derived algorithms apply different approaches based on ECG waveform analysis, including ECG signal decomposition through different wavelet transforms [19,20,21], variational mode decomposition [22], and empirical mode decomposition [23].

Methods based on the analysis of heart rate variability (HRV) and beat-to-beat (RR) interval time series [24,25] include temporal and spectral features, time frequency-based parameters, and some common complexity measures. Moreover, ECG-derived respiratory (EDR) signals obtained from breathing modulation on the T and R wave amplitudes are also combined with heart rate to reliably detect sleep apnea. Studies carried out by Penzel et al. [26], de Chazal et al. [27], and Méndez et al. [28] represent some examples that use features extracted from these EDR signals. The above methods rely on the fact that during apnea, the muscles of the respiratory system increase the mechanical effort to overcome the obstruction, thus affecting the surface ECG. This additional respiratory effort can impact other peripheral systems such as the cardiovascular system. In general, the patient’s respiratory information can be extracted from the fluctuations observed in the RR interval time series, namely HRV, and from the morphological changes caused by the respiratory modulation, by analyzing the EDR signals [29]. However, despite their proven higher accuracy, most algorithms using ECG signals are based on segmented intervals or epochs, most typically 1 min in duration, and many handcrafted features to train the models and perform the prediction task in an offline fashion. Although the ideal algorithm would involve automatic ECG feature extraction along with the use of shorter segments [30], even of varying size, the development of simpler models with finer time resolution could be a valid starting point for classifying shorter epochs or even a single apnea episode, as compared to the longer periods commonly used in epoch-based detection approaches.

The aim of this study was to estimate the occurrence probability of apnea episodes based on the effects caused by airway obstruction on various ECG markers. A preliminary version of this work has been reported in [31]. Specifically, we exploited the subtle changes that occur in heart rate and morphology-based ECG markers during individual episodes of apnea by using an experimental rat model of OSA. To this end, beat-to-beat time series of the aforementioned markers, together with apnea occurrence information, were used to train low-order dynamic Bayesian networks (DBNs) able to accurately detect short apnea-related epochs of only 15 s.

## 2. Materials and Methods

### 2.1. Population Data

Five healthy male Sprague–Dawley rats (mean weight: 437 ± 27 g) were intraperitoneally anesthetized with urethane (1 g/1 kg). The rats were connected to a system that controls a nasal mask with one tube open to the atmosphere and another connected to a positive pressure pump, thus preventing the animal from rebreathing. OSA episodes were simulated by obstructing the airways in the tubes by using electrovalves controlled by BIOPAC Systems (Goleta, CA, USA) equipment and its associated software. The electrodes and nasal mask were placed on the rats after the animals had been anesthetized and shaved.

### 2.2. OSA Model

Three recurrent apnea sequences were induced for 15-min intervals, preceded and followed by 15-min periods of normal breathing. A frequency (*F*) of 20, 40, or 60 apneas/hour was used for each sequence, simulating different levels of severity, while the duration of the apnea episodes was fixed at 15 s. The order of the apnea sequences as a function of *F* was randomized in each animal during the experiment. However, the number of sequences with the same *F* was evenly distributed within the population. Specifically, a total of 150 apneas of 15 s were provoked among all rats (30 apneas per rat), distributed over 15 separate recordings of 15 min each (three recordings per rat).

Several physiological signals were acquired with the BIOPAC system during spontaneous breathing and during the simulated apneas. These included respiratory flow, respiratory pressure, and electrocardiogram (ECG) signals (leads I and II) [32].

### 2.3. ECG Data Processing

The ECG signals were originally sampled at 1250 Hz, but then resampled at 10 kHz before further processing. This was performed to improve the time resolution for R-peak detection, since the heart rate range is significantly higher in rats (300–500 bpm) compared to humans [33]. The subsequent preprocessing steps included baseline drift attenuation via cubic spline interpolation [34], fourth-order bidirectional Butterworth low-pass filtering at 45 Hz to remove power-line interferences (50 Hz) and high-frequency noise, and, finally, automatic QRS complex detection [35] followed by a visual inspection to exclude noisy beats in noisy ECG segments.

#### 2.3.1. Heart Rate Periods

The final locations of the R-wave peaks were determined from the nonfiltered (low-pass) ECG signals based on previously detected QRS complexes, and used to generate the RR interval time series analyzed in this study.

#### 2.3.2. Morphology-Based ECG Markers

To enhance OSA detection, several ECG markers related to ventricular depolarization were also evaluated on nonfiltered ECG signals, thus providing relevant information about breathing influence on ECG waves morphology. These markers included the amplitude of the R and S waves (Ra, Sa), the difference between Ra and Sa (RSd), and the upstroke and downstroke slopes of the R wave, denoted by US and DS, respectively. US and DS were obtained as a result of fitting two straight lines over the ECG signal in specific segments within the QRS complex. Further details about the calculation of the QRS slope are described in [36].

The previously described markers were also used to obtain a combined marker by using principal components analysis (PCA). In brief, the beat-to-beat time series from the depolarization markers, Ra(t), Sa(t), DS(t), US(t), and RSd(t), were entered as inputs in the PCA algorithm. Then, a new signal projected onto the first component, defined here as QRSpca(t), was obtained to emphasize the apnea-related influence on the ECG. Finally, all single and combined marker time series are fed into the DBNs as described below.

### 2.4. Bayesian Networks Basics

Bayesian networks (BNs) are a type of probabilistic graphical model (PGM) representing the conditional independences among random variables with directed acyclic graphs (DAGs) [37,38]. BNs that can manage both discrete and continuous variables simultaneously are known as the so-called hybrid BNs, while those BNs containing only discrete or continuous nodes are called discrete BNs and Gaussian BNs (GBN), respectively. Each node in the DAG has an associated conditional probability distribution (CPD) that defines the probability distribution of the node given its parents in the DAG. In general, for a BN with *N* variables $X=\{X_{1},\ldots ,X_{N}\}$, the joint distribution factorizes as

(1) $$P\left(X\right)=\prod_{i=1}^{N}P\left(X_{i}|pa\left(X_{i}\right)\right),$$

where $pa(X_{i})$ denotes the configuration of the set of parents of $X_{i}$ in the network. Note that this factorization enforces several conditional independence statements. For instance, any variable in a Bayesian network is conditionally independent of the other variables of the model given its Markov blanket (mb), which is denoted as $X_{i}⊥X|mb\left(X_{i}\right)$ for short. Specifically, in a hybrid BN, the models are constructed as a set of conditional linear Gaussian (CLG) distribution models [39]. Here, discrete nodes are not allowed to have continuous parents. Likewise, the conditional distribution of each discrete variable $X\in X_{D}$ given its parents is a multinomial, while the conditional distribution of each continuous variable $Z\in X_{C}$ with discrete parents $Z_{D}\subseteq X_{D}$ and continuous parents $Z_{C}\subseteq X_{C}$, is given by

(2) $$f\left(z|pa\left(z\right)=\left\{z_{D},z_{C}\right\}\right)=N\left(z;\alpha \left(z_{D}\right)+\beta {\left(z_{D}\right)}^{T}z_{C},\sigma^{2}\left(z_{D}\right)\right)$$

for all $Z_{D}\subseteq X_{D}$ and $Z_{C}\subseteq X_{C}$, where α and β are the coefficients of a linear regression model of *Z* given its continuous parents. Note that this model can differ for each configuration of the discrete variables $Z_{D}$. After fixing any configuration of the discrete variables $X_{D}$, the joint distribution of any subset of the continuous variables $X_{C}$ is a multivariate Gaussian.

### 2.5. Dynamic Bayesian Networks

The obstructive apnea episodes affect several physiological variables from different interacting systems including the cardiac, respiratory, and neural systems. These variables present different responses with regard to amplitude variations and time durations, which may help to detect the apnea occurrence if their relationships can be modeled. In our study, these variables are defined as univariate time series that affect one another via unknown relationships. Therefore, we aim to model these relationships by using a DBN that combines both continuous and categorical variables, to approximate their temporal interactions during both normal respiration and apnea episodes.

Usually, DBNs assume that the underlying process they model represents a first-order Markovian, indicating that future states are independent of the past, given the present. However, in many biological processes, this particular assumption needs to be relaxed to include certain past information to improve model prediction of such future states.

In DBN, the time is discretized into slices for a given period. This period is usually determined by the frequency of the collected signals. For each time slice, there is a static BN that has parents in the previous slices in addition to the actual intraslice parents. In our study, each time slice is associated with each heartbeat occurrence. Here, the joint probability distribution accounts for all time slices from a certain time *T*,

(3) $$p\left(X_{0:T}\right)=p\left\{X_{0},X_{1},\ldots ,X_{T}\right\}=p\left(X_{0}\right)\prod_{t=0}^{T-1}p\left(X_{t+1}|X_{0:t}\right),$$

where $X_{t}=\left\{X_{1t},X_{2t},\ldots ,X_{nt}\right\}$ represents all the nodes in a time slice *t* for t=0,1,…,T. In this equation, it is required that all the previous time slices be taken into account to calculate the product. This can be simplified by using the Markov assumption [40]. On the other hand, the Markovian order defines the number of time slices required to confidently assume that the present is independent of the past. By applying a first-order Markovian network, the resulting joint probability distribution will be

(4) $$p\left(X_{0:T}\right)=p\left(X_{0}\right)\prod_{t=0}^{T-1}\left(X_{t+1}|X_{t}\right).$$

To compute the joint density distribution over time, the nodes in the current time slice and the parent nodes in the previous time slice only have to be considered. On the other hand, it is commonly assumed that the structure in a DBN is homogeneous over time, meaning that the network structure does not change with *t*. Because of this, the network structure including the intra- and interslice arcs and the parameters are replicated for each unrolled time step, which also means that only interslice arcs are permitted from t−1 to *t*. Increasing the Markovian order implies more arcs appearing from earlier lags to the present and thus a greater complexity when learning the network structure and its parameters.

#### 2.5.1. Structure Learning of DBN

The structure of the DBNs can be learned in two steps: (1) the intraslice arcs (static structure) of the network are learned with the max–min hill-climbing (MMHC) algorithm, (2) followed by learning the interslice arcs (transition structure). MMHC is a hybrid learning method that searches for possible network structures with a local search and then directs the arcs and scores the networks with the Bayesian information criterion (BIC) [41]. The local search is performed around each node to find its potential parents and children (i.e., its Markov blanket), which makes it conditionally independent of the other nodes. Specifically, a modified version of the MMHC algorithm named dynamic MMHC (DMMHC) was applied [42], in which the Markovian order of the network can also be modified in order to adjust the autoregressive order as needed.

Since the DBNs created here are CLG, the following assumptions are made: (a) discrete nodes have a multinomial distribution, (b) continuous nodes with no discrete nodes as parents have a normal distribution, (c) continuous nodes with at least one discrete node as a parent have a mixture of normal distributions, (d) all normal distributions in each mixture have a separate mean and variance, with each component having an independent set of parameters, and (e) discrete nodes can have only discrete nodes as parents, while continuous nodes can have both continuous and discrete parents [43].

#### 2.5.2. Parameter Learning in DBN

Once the network structure is learned, the next step consists of estimating the network parameters for each node. This step is performed based on the maximum likelihood estimator (MLE), whose specific form depends on the parents having each node and the assumed distribution for each variable. For continuous nodes with only continuous parents, the parameters of the local distribution are the regression coefficients associated with the parents and the standard deviation (SD) of the residuals [43]. In the case of continuous nodes with at least one discrete parent, the local distribution is modeled with a mixture of linear regressions by fitting a separate linear model for each data subset associated with the different values contained in the discrete parent. Finally, for discrete nodes, the conditional probabilities given their parents are estimated by using empirical frequencies. The same parameter-learning approach can be applied to DBNs once the structure is learned.

#### 2.5.3. Inference in DBN

After learning the structure and parameters of a DBN, it is possible to make inferences about any unobserved nodes or system states by providing some evidence to the network. For example, we can predict the most likely state of the system (i.e., whether there is an apnea episode or not) over the current interval. The evidence provided by past time slices should be used to predict the next time slice for a given node, either continuous or discrete, but also to estimate the actual value for some unknown parameter. In the case of Markovian orders higher than one, the prediction task consists of providing some evidence of the observed nodes for all time slices except the current one, and predicting the state of the desired nodes at time *t*,

(5) $$p(X_{i,t+\Delta }|Y_{:,0:t}),$$

where $X_{i,t+\Delta }$ represents the predicted variable at time t+Δ, and $Y_{:,0:t}$ represents all the evidence from t=0 to *T*. There are other special cases of interest such as the filtering, $p(X_{i,t}|Y_{:,0:t})$, and the fixed lag smoothing, $p(X_{i,t-\tau }|Y_{:,0:t})$, which are particularly considered in this study [44]. In such cases, $X_{i,t}$ and $X_{i,t-\tau }$ represent the *i*th hidden variable at time *t* and t−τ, respectively.

### 2.6. Data Preprocessing

**Figure 1:**
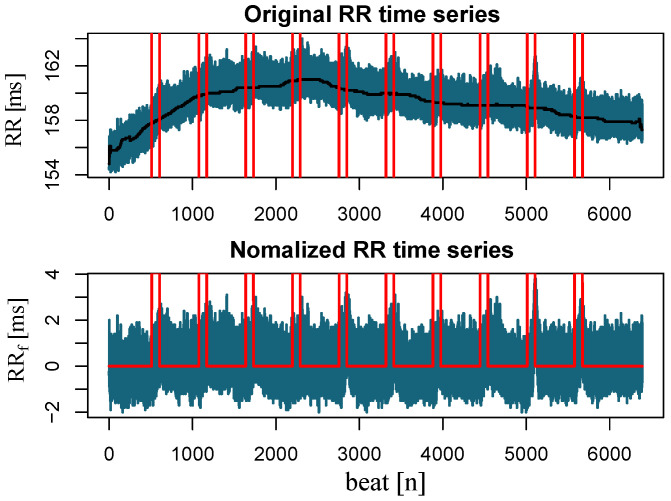
The RR time series (in blue) before (**top**) and after (**bottom**) removing the trends and slow oscillations (in black). Red vertical lines mark the time instant of apneas onset and end.

All time series TS(t) evaluated for our study were preprocessed before feeding into the DBN. For each marker, $TS_{f}\left(t\right)$, a detrended version was obtained by removing a 20th-order polynomial trend resulting from the original time series to attenuate slow oscillations that affect the detection rate and overall performance of the models. Since we are only interested in detecting apnea episodes, we do not care about the actual amplitude of the different markers, which can vary substantially between recordings. Figure 1 shows an example of this detrending process, emphasizing the effect caused by the apneas.

### 2.7. Model Training and Validation

#### 2.7.1. Univariate Conditional Lag-Based Models

To train the DBNs, we started with the first-order Markovian model (L = 1) by using only one time series $TS_{f}\left(t\right)$ at a time ($TS_{f}$ = {RR,Ra,Sa,DS,US,RSd,QRSpca}) in combination with the available apnea information, Ap={1,0}. Subsequently, the Markovian order was increased up to L = 15 to obtain more complex but better predictive models.

#### 2.7.2. Multivariate Conditional Lag-Based Models

**Figure 2:**
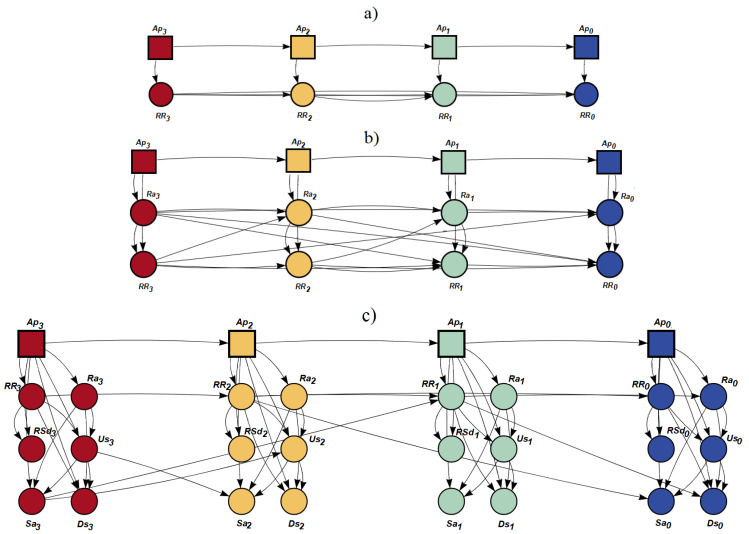
Examples of dynamic Bayesian networks obtained using four time slices (L = 3). (**a**) Only the RR time series. (**b**) The combination of Ra and RR time series. (**c**) All the ECG parameters (Ra, Sa, RSd, Us, Ds) combined with the RR time series.

A strategy similar to that described above was followed to train DBNs with more than one marker. Specifically, all paired combinations between the RR time series and the remaining ECG markers were used as input data for DBNs. In these cases, the apnea-detection rate can be significantly improved at the expense of more complex networks in terms of total number of nodes and arcs but with smaller orders. Finally, a model including all ECG markers together (excluding QRSpca) and the RR time series was also trained and compared to all previous simpler models. Figure 2 shows three examples of DBNs obtained for different combinations of parameters and fixing the lag value to L = 3, where the leftmost slice is the earliest ($t_{3}$) and the rightmost slice represents the present time ($t_{0}$).

The models were trained by using each 15-min recording of recurrent apnea simulated in each rat. This approach makes it possible to find the sequence that provides the best predictive model for a given animal. Model quality was assessed during model training by using both RMSE and Bayesian information criterion (BIC), which was also used to select the optimal lag for each network.

#### 2.7.3. Model Assessment

With the DBNs trained in each animal and sequence, the next step was to recursively compute the filtering density $p(x_{t}|y_{1:t})$, where *y* can be either the RR, any ECG parameter, or any combination of markers, while *x* represents the apnea occurrence at time *t*. The resulting conditional probability time series was then smoothed by using a 40-beat moving average filter to reduce potential spurious peaks.

**Figure 3:**
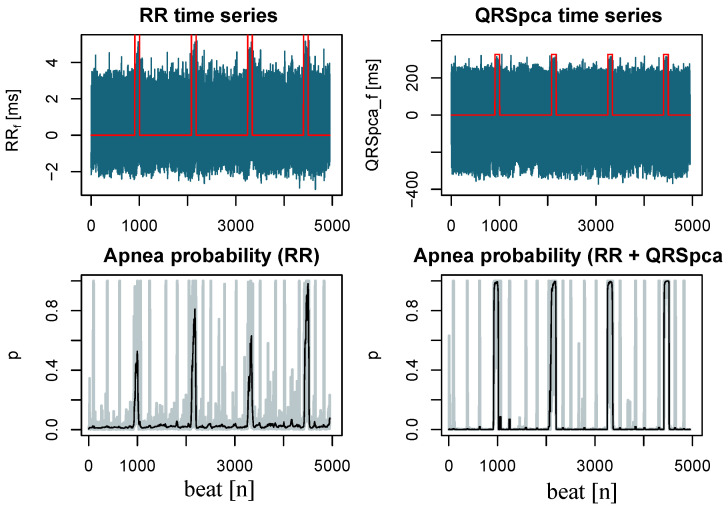
(**Top**) Normalized RR and QRSpca time series (blue color) and apnea occurrence (red color) for a particular segment of four apnea episodes. (**Bottom**) Apnea probability estimates using a DBN with L = 10, before (gray color) and after smoothing (black color) using only the RR marker, and when adding the QRSpca marker.

An example of this methodology is shown in Figure 3 for a particular segment of several apnea episodes. In this figure, the normalized time series of the RR and QRSpca markers are plotted with the actual apnea information in the top graphs, while the estimated apnea probability time series is shown before and after the smoothing step in the bottom graphs. Note that during apneas, the smoothed probability peaks are significantly larger and wider when using two parameters compared to the univariate approach. This implies a significant improvement in detection sensitivity.

The smoothed probability signal was then split into 15-s segments, or epochs, from which the RMS values were calculated. These RMS values were used to determine whether a segment was classified as normal or apneic. Finally, performance metrics including sensitivity (Se), specificity (Sp), accuracy (Acc), and area under the ROC curve (AUC) were calculated for all models as follows.

- Ideal record-specific analysis: DBNs were trained and tested on each 15-min recording of recurrent apnea (15 recordings of 60 epochs each) available for all rats. Performance measures were first averaged for each individual rat and then averaged across all rats.
- Record-specific analysis: DBNs were trained on the first recording of 15-min from each rat, and tested on the remaining two recordings from the same rat. The results from the testing phase were then averaged among all rats.

Finally, the average performance metrics were considered to select the optimal threshold and lag of the final network configuration for apnea detection.

## 3. Results

### 3.1. Exploring the Optimal Order Range for the DBNs

**Figure 4:**
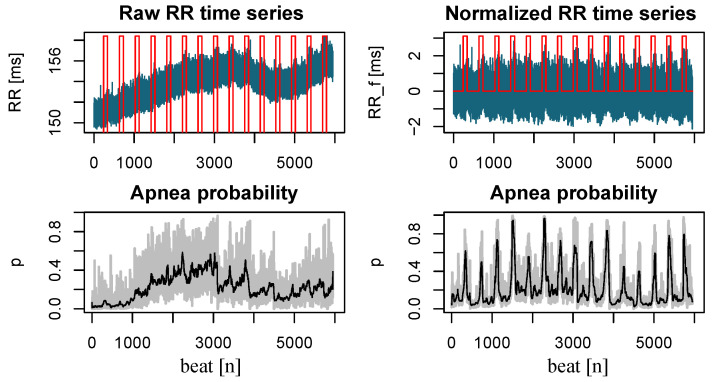
(**Top**) Original and normalized RR (blue color) and apnea occurrence (red color) for a particular sequence of 15 episodes. (**Bottom**) Apnea probability estimates (gray, raw values; black, average values) before and after the normalization process.

Figure 4 represents an example of the RR time series before and after removing the trend for a particular sequence of 15 apneas. As can be seen, several peaks are clearly observed on the smoothed probability signal (right side, bottom panel), corresponding to the timing of the occurrence of the apneas. However, the smoothed signal obtained before normalization (left side, bottom panel) follows a similar trend to that of the RR time series, significantly attenuating or masking the apnea-related peaks analyzed in further steps.

**Figure 5:**
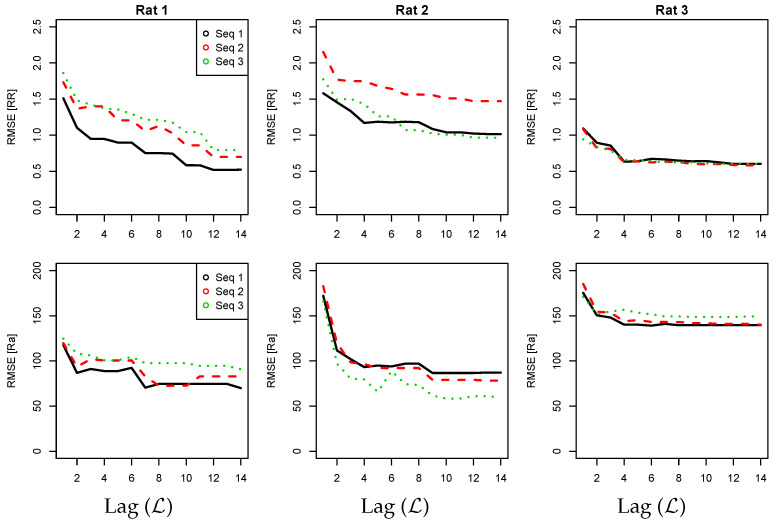
RMSE values as a function of the Markovian order when training DBNs in three different recordings for RR (**top**) and Ra (**bottom**) markers. Each color represents one particular sequence/recording of 15 min within the same rat having a different number of apnea episodes.

Regarding the optimal order range, Figure 5 illustrates the RMSE values obtained as a function of the network order for the RR and Ra markers in three particular recordings. For both markers, the RMSE values for the middle and right columns decrease rapidly from L = 1 to L = 5 and then remain at similar values up to L = 15. However, a slightly different pattern is observed for the example in the left column, where the RMSE values decrease slowly up to L = 10. In all cases, a very similar pattern is reflected for the three apnea sequences of each recording. Notably, depending on the rat analyzed, the sequences with smaller RMSE values along different order values may suggest the best one to train the final record-specific model. Several potential factors may affect the overall quality of the trained models, in particular the noise and the number of apnea episodes simulated in the time series.

### 3.2. Comparing Univariate Models: Heart Rate Fluctuations vs. Morphology-Based ECG Models

Having explored the suitable range of values for the network order in univariate models, the next step consists of training and testing DBNs by using each individual marker. Table 1 summarizes the results obtained during model assessment for all univariate models by using two different orders, L={5,15}.

From this table, it is clearly observed that the RR marker outperformed the results obtained for all morphology-based ECG markers in terms Se and Sp. In the case of L = 5, the Se value for RR doubled those corresponding to the ECG markers. When using L = 15, the Se values increased by approximately 20–35% among the different ECG markers and about 30% for the RR marker. However, QRSpca, Ra, and Us presented Sp and Acc estimates similar to those of RR. When testing the networks in the record-specific analysis by using the sequences not seen during training, the performance metrics decreased overall. In this case, the US resulted in the best marker with Se = 43.3%, Sp = 76.8%, and Acc = 74.7%.

### 3.3. Multivariate Models: Combining ECG Parameters with RR Interval

**Figure 6:**
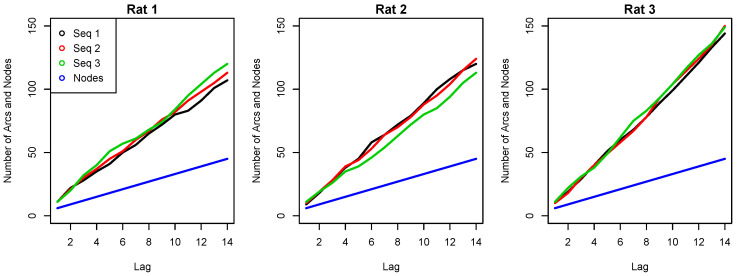
Complexity of the dynamic Bayesian networks as a function of lag (L) in three different recordings for the RR + Ra combination. The blue line represents the number of nodes involved while the other colors indicate the total number of arcs obtained from the networks’ structure in each sequence/recording.

Each individual morphology-based ECG marker was included as an additional input data to obtain different paired combinations. For instance, Figure 6 shows the evolution of the networks’ structure size for the combination of RR and Ra as a function of the network order. An almost linear increase in the total number of arcs is observed as the Markovian order increases. For the three examples shown in the figure, remarkable differences arise from L>4, but the smaller orders are quite similar. Note, however, that the number of nodes remains equal in the three sequences for all cases, while the number of arcs can vary significantly depending on the analyzed recording.

**Figure 7:**
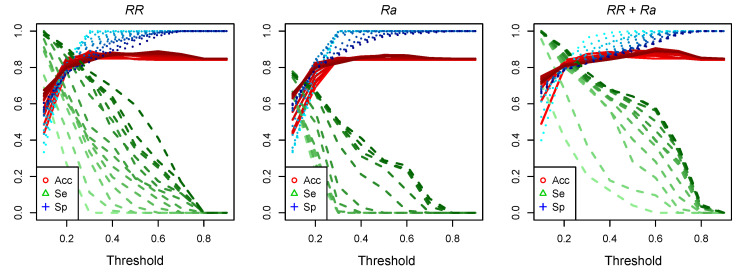
Performance metrics as a function of threshold obtained for Bayesian networks trained with individual (**left** RR, **middle** Ra) and combined (**right** RR + Ra) markers. Color intensity represents the lag (L = 1:15), where the lighter tones refer to smaller values.

Figure 7 (left and middle panels) shows the estimates of Acc, Se, and Sp obtained for two univariate models using RR and Ra, and the corresponding paired combination (right panel) in the ideal record-specific analysis. Different lags and thresholds (δ = {0.1,0.2,…,0.9}) were tested for the apnea-detection task in each epoch against their RMS estimates. As expected, the larger the threshold δ, the smaller the Se values in favor of higher Sp estimates. Note that the Acc and Sp values increased more rapidly up to thresholds of 0.3–0.5, depending on the lag L, but then remained fairly stable for larger values of δ. The curves obtained for Se by using the Ra marker alone showed a worse performance compared to the RR marker, even for smaller δ and larger L values.

Although univariate RR-based models showed very good overall performance, smaller orders yielded similar results when combining the RR and Ra markers (see Figure 7, right panel). The latter required only an order of L≥ 3 to achieve performance metrics similar to those obtained with L = 10 by using the RR marker alone. Table 2 summarizes the average results obtained during model evaluation by using all paired combinations for the ideal record-specific analysis. In these cases, with L = 5, we obtained results that were comparable to those with L = 15, with DBNs that are significantly less complex. The estimates of the performance metrics ranged from 77.4–83.7% for Se, 78.5–80.2% for Sp, and 80.3–81.8% for Acc, with Sa and US showing the best results. The results obtained when testing the paired combinations in the record-specific analysis decreased overall for all metrics, ranging from 57.2–70.6% for Se, 69.0–81.5 % for Sp, and 70.7–82.1 % for Acc. In this analysis, the best markers to combine with RR were RSd with Se = 64.4%, Sp = 79.7%, and Acc = 79.3%, and DS with Se = 68.9%, Sp = 81.5%, and Acc = 81.3%.

Note that the QRSpca marker was also combined with the RR marker and had a similar performance to other combinations, but only in the ideal record-specific analysis. On the other hand, when all ECG markers were used together, the observed improvement in performance metrics was not relevant enough compared to the paired combinations with the RR marker. The performance metrics for this case in the record specific analysis for L = 5 were Se = 69.4%, Sp = 78.4%, and Acc = 78.8%.

## 4. Discussion

In this study, we proposed a predictive model to detect obstructive apnea episodes by using heart rate information and morphological features obtained from a single ECG channel. The proposed model, based on DBN, was implemented and trained with ECG data obtained during an experimental model of obstructive sleep apnea in rats, where several sequences of recurrent apneas were simulated in a well-controlled protocol. In this sense, we explored different combinations of parameters and network configurations in order to (1) minimize the amount of prior information used to train the network, and (2) determine the best combination of physiological variables that will become part of the final structure. This was achieved by optimizing the network order (i.e., the number of previous heartbeats considered or lag) and searching for the best combination of features and optimal threshold that provides the best detection performance. Specifically, we analyzed the probability signal provided by the network and classified each 15-s epoch as apneic or normal breathing. The nature of our experimental protocol allowed us to use such short intervals, unlike the conventional 1-min epochs used in many patient studies, as well summarized in [9]. The epoch length can be reduced if necessary, since the final DBN is composed of only five consecutive heartbeats.

Several findings can be highlighted from the results obtained. First, the higher the Markovian order (lag) used for the trained DBNs, the better the sensitivity estimates, at the expense of higher model complexity. However, beyond a certain order, usually above 10, the performance did not improve further, since the number of arcs increases exponentially (see Figure 6). In fact, larger orders showed worse performance metrics when testing the model on data not seen during training. Secondly, when searching for the best predictive variables, it was found that the RR marker alone outperformed any single morphology-based ECG marker, especially in terms of sensitivity values. However, paired combinations including the RR marker seemed to significantly reduce the network order compared to those networks using a single marker. This means that adding more diverse information to the network allows one to obtain more sensitive models with reduced past information, supporting the importance of multivariate models in prediction problems. Furthermore, when attempting to combine all ECG markers using PCA to further increase apnea-detection rates, no significant improvement in performance metrics was observed compared to the paired combinations. Similar results were obtained when all ECG markers were combined as input variables, but with more smoothed performance curves when threshold and order were varied. A possible reason for this could be that all morphology-based ECG markers were associated with the depolarization phase. In this regard, adding other markers from the ECG, such as repolarization markers, or from another physiological signal, could improve the overall performance.

Another relevant aspect worth mentioning is the normalization process applied to the time series before training the dynamic Bayesian networks. As demonstrated in the Methods section, this process increased the chances of detecting apnea episodes in some sequences particularly affected by the amplitude drifts observed in the parameters. On the other hand, this normalization could help to obtain a more generalized model suitable for all cases, regardless of animal and stage, instead of a personalized model for each record. An analysis of this particular step has been discussed in [45] for a human study.

Despite the fact that the overall detector performance was approximately 80%, we believe it is good enough considering the limited number of variables used to train the DBNs. Nevertheless, depending on the rat analyzed, the detection performance for the record-specific analysis was in some cases higher than 90%. However, only the first recording (one-third of the data) was used to train the model with respect to each particular rat; it was then applied to the following two sequences of 15 min. Preliminary analysis, not presented here, showed that training DBNs with later sequences could successfully predict apnea episodes from earlier sequences. In fact, for some animals, the performance measures were better with the model trained with a different recording than those obtained with the model trained on the same recording. This fact suggests the importance of selecting the most appropriate segment to train the record-specific models. It could be extrapolated, for example, to the best PSG recording among several, if available for the same OSA patient, as happens in the Apnea ECG database of the PhysioNet [46], leading to a more robust and personalized model for continuous monitoring of patients. In clinical studies conducted in OSA patients where the epoch duration was shorter than 60 s (i.e., 30 s, 40 s), the results obtained by the proposed methods were 70% [47] and 88% [48] of accuracy using 40-s epochs and involving 21 and 6 subjects, respectively. In the case of 30-s epochs, the accuracy obtained was 75% with 100 involved records [49] and 89% with 12 subjects [50]. The methods that achieved the highest scores were actually developed from one or more respiration-based signals instead of ECG channels.

Furthermore, the number of previous heartbeats used in the training phase was significantly small. Here, the largest order used (L = 15) sometimes does not exceed 3 s of the analyzed time series, due to the fast heart rhythm of the rats. Longer periods could be used if the original time series is resampled at a lower and fixed frequency, so that more information can be considered at once in the DBN. The latter might also reduce the noise observed in the raw probability time series of the network output. However, the end goal for future work should be a simple predictive network that may eventually be suitable for real-time applications in wearable or ambulatory monitoring devices.

Finally, many clinical studies performed in OSA patients have also used only ECG features to detect sleep apnea, even using a single channel [9]. These features typically include temporal and spectral markers derived from heart rate variability (HRV) analysis, as well as a variety of ECG markers derived from both depolarization and repolarization periods. The majority of these studies have used longer epochs, with 1-min epochs being the most common, to train complex multivariate models that typically run in an offline fashion. These longer epochs may contain multiple apneas of short duration and different types, making it difficult to detect and classify individual episodes and automatically estimate the Apnea–Hypopnea Index (AHI). The design of simpler models with a refined time resolution can be of interest for real-time scenarios. In particular, it can benefit their hardware implementation, thus having a great impact on home monitoring systems, wearable devices, and healthcare for OSA patients.

### Limitations

Our study had some limitations, especially the small sample size, the fixed apnea duration, and the single event type. Our experimental model simulated only obstructive apnea episodes of 15 s duration, which is not the real case in OSA patients. A future step could be to develop and test similar models by using PSG recordings from OSA patients. In particular, they should include a wide range of disease severity, event duration and type, and occur at nonperiodic intervals. Further work is needed to achieve comparable results in humans, where the detection and diagnosis of OSA is much more difficult than in well-controlled experimental models. This may involve redefining the model and testing it on other different ECG leads, including cardiac repolarization features and EDR signals, to achieve similar performance metrics.

## 5. Conclusions

The proposed methodology, based on dynamic Bayesian networks, allowed us to obtain record-specific models for apnea segment detection by using short 15-s epochs and a single ECG channel. Although implemented on experimental data from an OSA model, the methodology represents a first step toward the development of a real-time monitoring tool for OSA patients. Successful implementation of this tool on smart health devices could be used to detect and classify abnormal events over several nights instead of a single night, allowing the patient’s disease progression to be followed. In addition, it could also estimate the number of events per hour as a potential surrogate for the AHI.

## Figures and Tables

**Table 1:** Overall apnea detection performance obtained for the RR and ECG markers by using L={5,15} and threshold equal 0.2.

		L=5			L=15	
	Se	Sp	Acc	Se	Sp	Acc
Marker	(%)	(%)	(%)	(%)	(%)	(%)
RR	63.3	84.8	84.0	91.1	76.7	79.8
Ra	35.2	80.2	77.1	66.7	79.5	79.8
Sa	30.1	76.7	73.6	64.4	77.4	77.6
RSd	32.6	70.2	68.9	51.1	74.8	73.9
Us	29.6	75.8	72.7	65.2	79.0	79.1
Ds	31.1	68.4	67.3	50.4	74.0	73.2
$QRS_{PCA}$	40.0	80.1	77.6	63.7	79.2	79.0

**Table 2:** Overall apnea-detection performance obtained for RR and all paired combinations by using L={5,15}.

		L=5			L=15	
	Se	Sp	Acc	Se	Sp	Acc
Marker(s)	(%)	(%)	(%)	(%)	(%)	(%)
RR						
Training	63.3	84.8	84.0	91.1	76.7	79.8
Testing	80.6	68.6	70.5	67.8	69.6	69.2
RR + Ra						
Training	81.1	79.5	81.3	90.4	78.4	80.6
Testing	58.3	74.3	74.0	60.0	69.1	70.2
RR + Sa						
Training	81.9	80.1	81.8	85.6	77.2	79.1
Testing	64.4	69.7	70.7	55.0	74.5	73.7
RR + RSd						
Training	83.7	79.8	81.6	86.3	76.0	77.8
Testing	64.4	79.7	79.3	62.0	64.8	66.7
RR + Us						
Training	83.0	80.2	82.1	89.3	77.7	80.3
Testing	70.6	69.0	71.2	63.3	71.8	73.0
RR + Ds						
Training	79.3	80.1	81.6	85.2	76.5	78.1
Testing	68.9	81.5	81.3	56.7	75.1	74.5
RR + PCA						
Training	77.4	80.0	81.1	85.6	78.2	80.2
Testing	57.2	70.2	70.7	58.3	67.2	68.4

## Data Availability

The data presented in this study are available on request from the corresponding author.

## Abbreviations

The following abbreviations are used in this manuscript:

OSA	Obstructive sleep apnea
DBN	Dynamic Bayesian networks
AHI	Apnea–Hypopnea Index
HRV	Heart rate variability
PCA	Principal component analysis
RMSE	Root mean square error
ROC	Receiver operative characteristic
MAP	Maximum a posteriori
CP	Conditional probability
MLE	Maximum likelihood estimator
MMHM	Max–min hill-climbing
CLG	Connditional linear Gaussian
BIC	Bayesian information criterion
DAG	Directed acyclic graphs
CPD	Conditional probability distribution
PGM	Probabilistic graphical model
EDR	ECG-derived respiratory signal
PSG	Polysomnography
IH	Intermittent hypoxia
